# Endometriosis and Endometrial Cancer—Association Between Biological Mechanisms and Its Clinical Implications

**DOI:** 10.3390/jcm15082891

**Published:** 2026-04-10

**Authors:** Karolina Maria Marczuk, Mateusz Bartosz Mamala, Ewa Magdalena Szuster, Marek Murawski

**Affiliations:** 1 Faculty of Medicine, Wroclaw Medical University, 50-367 Wroclaw, Poland; karolina.marczuk@student.umw.edu.pl (K.M.M.); mateusz.mamala@student.umw.edu.pl (M.B.M.); 2 Clinical Department of Gynecologic Surgery and Oncology, Wroclaw Medical University, 50-367 Wroclaw, Poland; marek.murawski@umw.edu.pl

**Keywords:** endometriosis, endometrial cancer, chronic inflammation, estrogen dependence, molecular pathways, personalized medicine

## Abstract

Endometriosis and endometrial cancer are distinct gynecological conditions that share overlapping biological mechanisms with implications for clinical management. Endometriosis is a chronic, benign disorder characterized by the ectopic implantation of functional tissue lining the uterus, primarily affecting women of reproductive age. It commonly causes chronic pelvic pain, dysmenorrhea, dyspareunia, and infertility. The disease is marked by persistent inflammation, hormonal dysregulation, and alterations in cellular signaling, which mirror some neoplastic processes despite lacking malignant potential. Endometrial cancer is a malignant tumor of the uterine lining, most frequently diagnosed in postmenopausal women. Its incidence is rising due to aging, obesity, and prolonged estrogen exposure. Epidemiological studies suggest a modest increase in endometrial cancer risk among women with endometriosis. However, detection bias and metabolic confounders may influence this association. Both conditions share estrogen dependence, chronic inflammatory microenvironments, and dysregulated pathways such as PI3K/AKT/mTOR. Somatic mutations in genes, including PTEN and ARID1A, further underline molecular intersections. Clinical management is tailored to disease type and severity. Endometriosis therapy emphasizes stepwise hormonal treatment, multidisciplinary pain management, and surgery when indicated. Endometrial cancer management relies on staging, with particular emphasis on molecular classification and histopathology to guide surgery, radiotherapy, chemotherapy, hormone therapy, and immunotherapy in advanced cases. Emerging noninvasive biomarkers and precision medicine strategies may enhance diagnosis, monitoring, and targeted treatment in both conditions. Understanding their shared and divergent mechanisms aids risk stratification, individualized therapy, and improved quality of life. Further prospective studies are needed to optimize patient-specific management and translate mechanistic insights into clinical practice.

## 1. Introduction

Endometriosis is a chronic, benign gynecological disease characterized by the ectopic presence of tissue resembling the endometrium, most commonly involving pelvic structures and the peritoneal surface. The disease predominantly affects women of reproductive age. It is frequently associated with chronic pelvic pain, dysmenorrhea, dyspareunia, and impaired fertility. From a pathophysiological perspective, endometriosis is marked by persistent inflammatory activity, disturbances in hormonal regulation, and alterations in the cellular microenvironment that share certain biological features with neoplastic processes. However, the disease itself is not classified as malignant. Epidemiological evidence has demonstrated an association between endometriosis and an elevated risk of selected malignancies, particularly ovarian cancer, while its relationship with endometrial cancer has been investigated with inconsistent and sometimes conflicting results [1,2]. The most diagnosed gynecologic malignancy in developed countries is endometrial cancer—a malignant tumor arising from the endometrial lining of the uterus. Over recent decades, its incidence has increased worldwide, especially among postmenopausal women. This trend is primarily driven by an aging population, rising obesity prevalence, and prolonged exposure to hormonal risk factors. In contrast to endometriosis, which primarily affects women during their reproductive years, endometrial cancer is typically diagnosed later in life, with most cases occurring after menopause. A comprehensive understanding of both conditions and their potential interrelationships is essential for optimizing clinical management and improving the identification of populations at increased risk [3,4].

## 2. Epidemiology

### 2.1. Endometriosis

Endometriosis is a common gynecological disease affecting women of reproductive age worldwide. Although prevalence estimates differ because of variations in study design and diagnostic methods, approximately 190 million women are affected globally, with higher prevalence among those evaluated for gynecological symptoms of infertility [5]. Because diagnosis has traditionally relied on laparoscopic and histopathological confirmation, while noninvasive methods remain limited, endometriosis is frequently underrecognized and associated with substantial diagnostic delay [5,6,7].

In addition to its clinical burden, endometriosis has been investigated in relation to malignancy risk. Its association with ovarian cancer, particularly endometrioid and clear-cell subtypes, is well established, whereas the link with endometrial cancer remains less consistent [8]. Nevertheless, several large epidemiological studies have shown a modest but statistically significant increase in endometrial cancer risk among women with endometriosis [2]. This association should be interpreted with caution, as intensified gynecological surveillance in these patients may contribute to earlier or incidental detection of endometrial abnormalities and malignancies, thereby partly inflating the observed risk [2].

### 2.2. Endometrial Cancer

Endometrial cancer is the most common uterine malignancy and one of the most frequently diagnosed gynecologic cancers worldwide [9,10]. It is the leading gynecologic malignancy in developed countries, and its incidence has risen steadily in parallel with population aging and the increasing prevalence of obesity and metabolic syndrome [4]. Among women over 50, incidence has nearly doubled over the past three decades, whereas mortality rates have declined, likely owing to advances in early detection and treatment [3]. In 2021, uterine cancer, predominantly endometrial in origin, accounted for tens of thousands of new cases worldwide, with marked geographic variation reflecting demographic, lifestyle, and reproductive factors [11].

The disease primarily affects postmenopausal women, with a median age at diagnosis of 60–70. However, a minority of cases occur in younger individuals [4]. Major risk factors include obesity, metabolic syndrome, hypertension, prolonged unopposed estrogen exposure, nulliparity, early menarche, late menopause, insulin resistance, and inherited cancer predisposition syndromes [12]. Obesity is one of the most important modifiable determinants, with risk increasing consistently with body mass index [12]. Notably, insulin resistance and chronic adipose tissue inflammation may represent a common mechanistic link between endometrial cancer and endometriosis [13,14]. Obesity-associated hyperinsulinemia reduces sex hormone-binding globulin (SHBG) levels and increases estrogen bioavailability, while adipose tissue-derived proinflammatory cytokines promote a chronic inflammatory milieu [13,14]. Together, these metabolic and inflammatory pathways may support both ectopic endometriotic lesion growth and malignant transformation of the endometrium [13,14].

## 3. Pathophysiology and Shared Mechanisms

Notwithstanding that endometriosis and endometrial cancer are classified as benign and malignant disorders, respectively, they share a number of overlapping hormonal, inflammatory, and molecular features. These similarities have raised increasing interest in whether common biological mechanisms may contribute to their coexistence or sequential development in selected patients [15]. Rather than representing a simple association, the relationship between these conditions appears to involve a complex interplay between estrogen signaling, chronic inflammation, oxidative stress, dysregulated intracellular pathways, and alterations in genetic and epigenetic regulation [15].

### 3.1. Hormonal Dysregulation and Estrogen Dependence

A central shared feature of endometriosis and endometrial cancer is estrogen dependence. Endometriosis is characterized by local estrogen overproduction within ectopic lesions, driven by increased aromatase activity and impaired inactivation of estradiol due to reduced expression of 17β-hydroxysteroid dehydrogenase type 2 (HSD17B2). This results in sustained estrogenic stimulation that promotes lesion survival and proliferation [5,16]. In parallel, progesterone resistance further enhances the persistence of ectopic lesions by weakening antiproliferative and anti-inflammatory progesterone signaling [17].

Similarly, endometrial cancer, particularly the estrogen-dependent type I subtype, arises in the setting of prolonged exposure to unopposed estrogen, which promotes endometrial hyperplasia and subsequent malignant transformation [3,12]. This hormonal milieu is amplified in obesity, especially after menopause, when peripheral aromatization in adipose tissue becomes a major source of estrogen synthesis [3,12]. In addition, hyperinsulinemia may reduce SHBG levels, thereby increasing the bioavailability of circulating estrogens and further potentiating endometrial proliferation [18].

Taken together, these observations indicate that aberrant estrogen signaling is not only a shared endocrine feature of both diseases but also a biologically plausible bridge between ectopic lesion maintenance and endometrial carcinogenesis [19].

### 3.2. Chronic Inflammation and Oxidative Stress

Beyond endocrine dysregulation, chronic inflammation represents another fundamental intersection between endometriosis and endometrial cancer. Endometriotic lesions develop within a proinflammatory microenvironment characterized by elevated levels of cytokines such as interleukin 6 (IL-6), tumor necrosis factor-α (TNF-α), and prostaglandin E2 (PGE2), which promote angiogenesis, immune evasion, and resistance to apoptosis [13]. These inflammatory mediators contribute to lesion persistence and may also facilitate tissue remodeling and neuroangiogenesis, thereby linking inflammation with both disease progression and symptom severity [20].

Oxidative stress is increasingly recognized as an important companion mechanism in endometriosis. Recurrent bleeding within ectopic lesions, iron accumulation, and activated immune cells promote reactive oxygen species generation, which may enhance DNA damage, lipid peroxidation, and aberrant cellular signaling. This oxidative milieu may strengthen the inflammatory phenotype of endometriotic tissue and has been proposed as one of the mechanisms that could favor neoplastic change in susceptible contexts [21].

In endometrial cancer, chronic inflammation is frequently associated with obesity, insulin resistance, and adipose tissue dysfunction. Adipose tissue acts as an active endocrine organ, releasing proinflammatory adipokines and mediators that stimulate proliferation and oxidative stress within the endometrium [11,14,22]. These processes may contribute to genomic instability, altered cell survival, and malignant progression [11,14]. Reactive oxygen species may additionally modulate cancer cell survival, proliferation, and treatment response, indicating that oxidative stress is relevant not only to carcinogenesis but also to disease behavior [23].

Thus, chronic inflammation and oxidative stress should be viewed as interdependent drivers that may help to explain part of the biological overlap between endometriosis and endometrial cancer [23].

### 3.3. Molecular Pathways: PI3K/AKT/mTOR and Beyond

At the molecular level, both endometriosis and endometrial cancer exhibit dysregulation of signaling pathways involved in proliferation, survival, metabolism, and cellular plasticity. Among these, the PI3K/AKT/mTOR pathways appear particularly important. In both ectopic endometrial tissue and malignant endometrial cells, aberrant activation of this pathway supports enhanced proliferation, resistance to apoptosis, and metabolic reprogramming [2,20,24].

However, PI3K/AKT/mTOR should not be considered in isolation. Other signaling cascades, including MAPK, inflammatory NF-κB signaling, and Wnt/β-catenin-related pathways, also contribute to disease progression and may interact with estrogenic and inflammatory stimuli [25]. In endometriosis, these pathways are implicated in adhesion, invasion, angiogenesis, and lesion maintenance. In endometrial cancer, they participate in tumor growth, stemness, immune escape, and therapeutic resistance. Their crosstalk suggests that the common biology of both disorders is better understood as a network of interconnected signaling systems rather than as a single linear pathway [26,27].

This broader mechanistic perspective may be particularly useful for future risk stratification and for identifying therapeutic targets relevant to both endometriosis-associated proliferative activity and endometrial tumor progression [27].

### 3.4. Genetic and Epigenetic Alterations

Genetic and epigenetic alterations provide further support for biological convergence between endometriosis and endometrial cancer. Somatic mutations in genes such as PTEN, ARID1A, and PIK3CA, classically associated with endometrial and other gynecologic malignancies, have also been identified in endometriotic lesions [2,16,28,29]. These observations support the idea that endometriosis may, at least in some cases, represent a clonal proliferative disorder rather than a purely reactive condition [2,16,28,29].

At the same time, epigenetic dysregulation appears to be highly relevant in both diseases. Altered DNA methylation, histone modification, and non-coding RNA expression may influence steroid hormone responsiveness, inflammatory signaling, cell survival, and tissue remodeling [30,31]. In endometrial cancer, epigenetic dysregulation contributes to tumor initiation, progression, and molecular heterogeneity. It is increasingly being explored for biomarker development and therapeutic targeting [31].

Importantly, the coexistence of genetic driver mutations with epigenetic remodeling suggests that shared pathogenesis may involve both stable genomic alterations and reversible regulatory mechanisms. This may help explain why some lesions display proliferative and invasive behavior without undergoing full malignant transformation [30,31,32].

### 3.5. Epithelial–Mesenchymal Transition

Epithelial–mesenchymal transition (EMT) represents another potentially important shared mechanism. In endometriosis, EMT has been implicated in the acquisition of migratory and invasive properties by ectopic endometrial cells. Loss of epithelial characteristics together with increased mesenchymal traits may facilitate implantation, fibrosis, lesion persistence, and resistance to local regulatory signals. EMT in endometriosis appears to be influenced by inflammatory mediators, hypoxia, and TGF-β-related signaling [33,34].

In endometrial cancer, EMT is closely associated with invasive growth, metastatic dissemination, and treatment resistance. Altered expression of markers such as E-cadherin, β-catenin, vimentin, and EMT-associated transcription factors may reflect a more aggressive biological phenotype. Notably, EMT may also function as a mechanistic bridge between estrogen signaling, inflammation, and intracellular pathway activation, thereby integrating several of the major processes discussed above [35,36].

Although the biological consequences of EMT differ between benign chronic disease and a malignant tumor, its presence in both settings reinforces the idea that endometriosis and endometrial cancer share aspects of cellular plasticity that are clinically and mechanistically relevant [37,38].

### 3.6. Converging and Diverging Mechanisms

Despite these similarities, important biological distinctions remain. Endometrial cancer is defined by invasive growth, metastatic potential, and broader genomic instability, whereas endometriosis generally lacks the full hallmarks of malignancy [39]. Although endometriosis may involve multiple pelvic and extrapelvic locations, it does not exhibit true metastatic spread [39]. The mechanisms that limit the transition from chronic estrogen-dependent inflammation to overt malignant transformation remain incompletely understood and likely involve additional constraints related to immune surveillance, stromal context, and the degree of accumulated genomic damage [40,41,42].

Overall, endometriosis and endometrial cancer share a complex network of hormonal, inflammatory, oxidative, and molecular mechanisms that may explain their epidemiological association in selected populations [15]. A clear understanding of both convergent and divergent pathways is essential for improving biological risk stratification, refining surveillance concepts, and identifying therapeutic targets relevant to both diseases.

## 4. Association Between Endometriosis and Endometrial Cancer

The biological overlap between endometriosis and endometrial cancer described in the previous section has led researchers to examine whether these shared mechanisms are reflected at the epidemiological level [24]. Numerous observational studies have examined the incidence of endometrial cancer among women with a history of endometriosis, although the outcomes have varied. Several large cohort studies and meta-analyses indicate that individuals with endometriosis face a slightly higher risk of developing endometrial cancer compared to the general population. A pooled analysis of observational studies reported a relative risk of approximately 1.66 (95% CI 1.15–2.41) for endometrial cancer in patients with endometriosis. However, substantial between-study heterogeneity was observed, indicating that the magnitude of this association varies across populations and study designs [2]. In contrast, other systematic reviews have emphasized that the epidemiological link between endometriosis and endometrial cancer is less consistent than the well-established association with ovarian cancer [24]. Other systematic reviews have emphasized that, although some studies suggest an elevated risk, the overall evidence remains methodologically limited by heterogeneity, bias, and incomplete control of temporality and confounding. In particular, Kvaskoff et al. stressed that many studies addressing cancer risk in endometriosis carry a moderate to critical risk of bias, which weakens causal inference [5]. Several studies have emphasized that the observed association between endometriosis and endometrial cancer may be substantially influenced by confounding factors such as body mass index, parity, age, and hormonal exposures, limiting causal interpretation [5,43,44]. Population-based cohort studies provide additional, although nuanced, evidence. Registry-based analyses from different geographic regions have reported higher incidence rates of endometrial cancer among women with endometriosis, particularly when endometriosis was diagnosed later in reproductive life [45]. A recent propensity score-adjusted real-world study by Farolfi et al. also found an increased risk of endometrial cancer in women with endometriosis. However, this type of evidence still does not by itself establish direct biological progression from one disease to another [29,46]. Nevertheless, these findings are sensitive to follow-up duration, diagnostic accuracy, and case definition of endometriosis [5]. An important methodological issue in interpreting these data is detection and surveillance bias. Patients with endometriosis are more likely to undergo long-term gynecological follow-up, including repeated imaging and clinical examinations, which may increase the likelihood of detecting asymptomatic or early-stage endometrial cancer [2]. Consequently, part of the reported increase in endometrial cancer risk may reflect heightened diagnostic intensity rather than a true biological progression [24]. In addition, the epidemiological association should be interpreted in the context of potential confounding factors. Obesity and metabolic syndrome are among the strongest established risk factors for endometrial cancer. They may partially account for the modest increase in risk observed in some population-based studies. Hormonal and reproductive factors, including prolonged estrogen exposure, nulliparity, age, and the use of hormonal treatment, may also differ across study populations and influence the observed association. Because the reported effect size is relatively small, residual confounding and differences in clinical surveillance may substantially affect the estimated risk [2]. The distinction is particularly important: a modest epidemiological association does not necessarily imply that endometriosis commonly undergoes direct malignant transformation into endometrial cancer. Current evidence supports the view that the association between these diseases is more likely explained by partly shared hormonal, inflammatory, and metabolic risk environments than by a frequent linear progression from ectopic endometriotic lesions to uterine malignancy. This interpretation is also consistent with large reviews showing that the strongest and most reproducible cancer association in endometriosis concerns ovarian endometrioid and clear-cell carcinomas rather than endometrial carcinoma [20,47,48]. A discussion of atypical endometriosis further helps clarify this distinction. Atypical endometriosis is characterized by cytologic and/or architectural atypia within endometriotic tissue. It has been recognized as a potential precursor lesion, particularly for endometriosis-associated ovarian cancer, most notably the endometrioid and clear-cell ovarian subtypes. The strongest pathological and molecular evidence for a true precursor relationship, therefore, concerns the ovary, not the endometrium. The recent systematic review by Capozzi et al. describes atypical endometriosis as an intermediate entity between typical endometriosis and endometriosis-associated ovarian cancer, while Ioannidou et al. similarly emphasize that malignant transformation of endometriosis, when it occurs, is predominantly linked to ovarian tumors [29,49]. For this reason, although atypical endometriosis should be acknowledged as a clinically important precancerous condition, it should not be presented as direct evidence of a frequent precursor pathway to endometrial cancer. Rather, its relevance in the present review is conceptual: it demonstrates that under particular molecular and microenvironmental conditions, some endometriotic lesions may acquire premalignant features, while the epidemiological association with endometrial cancer remains comparatively weaker, less uniform, and more vulnerable to bias and confounding [5,29,49]. Taken together, current epidemiological evidence supports a slight association between endometriosis and endometrial cancer. However, this relationship is neither strong nor uniform across studies and should be interpreted with caution [2]. The modest magnitude of risk suggests that shared hormonal and inflammatory pathways, along with common metabolic risk factors and surveillance effects, are more likely explanations than direct malignant transformation of endometriosis [24,50].

Clinical and biological characteristics of endometriosis and endometrial cancer are illustrated in Table 1.

## 5. Clinical Management

The clinical management of endometriosis and endometrial cancer reflects the fundamental biological distinction between a chronic, estrogen-dependent inflammatory condition and a malignant disease with invasive potential [16]. Even so, the two conditions should not be discussed as entirely unrelated from a therapeutic perspective. Both are shaped by estrogen signaling, inflammatory mediators, angiogenesis and intracellular growth pathways, including PI3K/AKT/mTOR-related signaling [3,13,16]. What differs is not the existence of these pathways, but the clinical purpose for which they are targeted. In endometriosis, treatment is primarily directed toward pain relief, suppression of lesion activity, reduction in recurrence and preservation of fertility and quality of life [55,56]. In endometrial cancer, by contrast, treatment is guided by oncologic objectives, including disease eradication, recurrence prevention and survival improvement, with management determined by stage, histotype and molecular subtype [57,58,59,60].

This difference in therapeutic intent is essential when interpreting why biologically similar pathways occupy very different positions in treatment algorithms. A pathway that represents a rational target in both diseases does not necessarily justify the same therapeutic strategy, because acceptable toxicity, treatment duration and reproductive considerations differ profoundly between benign chronic disease and cancer [61,62,63]. For that reason, clinical management should be viewed not only through the lens of treatment modality, but also through the lens of therapeutic target and treatment goal. This perspective is particularly useful when comparing endocrine therapy, anti-inflammatory approaches, antiangiogenic strategies and inhibitors of PI3K/AKT/mTOR signaling across both conditions. It also helps explain why some cancer-derived therapeutic concepts may inform future endometriosis research, while still remaining unsuitable for routine use in a benign disease because of toxicity, cost, or incompatibility with long-term treatment and reproductive planning [61,64,65,66].

### 5.1. Management of Endometriosis

The management of endometriosis is centered on long-term symptom control, the suppression of disease activity, and the reservation of fertility, with treatment tailored to disease phenotype, symptom burden, reproductive plans, and previous treatment response [55,56]. In symptomatic patients without immediate reproductive goals, current guidelines continue to support medical therapy as the first-line approach [55,56]. This preference reflects not only the chronic nature of the disease, but also the fact that endometriosis is usually managed as a long-term estrogen-dependent inflammatory disorder rather than as a lesion burden requiring eradication at any cost.

Hormonal therapy remains the foundation of nonsurgical treatment because it reduces the estrogenic stimulation of ectopic endometrial tissue and thereby limits proliferation, bleeding and inflammatory activity [55,56]. Commonly used options include combined oral contraceptives, progestins such as dienogest, and GnRH agonists or antagonists [55,56,67]. Progestins remain especially relevant in long-term treatment because they promote decidualization and atrophy of lesions while maintaining acceptable tolerability for extended use [67]. GnRH antagonists have further expanded the therapeutic landscape by enabling rapid, dose-dependent suppression of estradiol without the flare effect seen with GnRH agonists [68]. Current ESHRE guidance places GnRH antagonists as a second-line option when contraceptives or progestogens are ineffective or poorly tolerated [55].

This endocrine approach also illustrates an important principle developed further in the comparative discussion below: although endometriosis shares several molecular pathways with endometrial cancer, endocrine suppression occupies a broader place in endometriosis because treatment must usually be compatible with prolonged administration, symptom control and reproductive planning rather than oncologic endpoints [55,56,69,70,71]. For the same reason, therapies that are biologically rational but associated with greater toxicity have remained limited or investigational in endometriosis despite targeting relevant pathways.

Aromatase inhibition is one example. Because local estrogen biosynthesis has been demonstrated in endometriotic lesions, aromatase inhibitors provide a mechanistically attractive strategy, especially in refractory disease [72,73]. However, they are not considered first-line therapy, largely because of hypoestrogenic adverse effects, possible effects on bone mineral density and limited suitability for long-term use in young women [72,73]. Similarly, the PI3K/AKT/mTOR pathway and angiogenic signaling are increasingly recognized as relevant to lesion survival, fibrosis, progesterone resistance and vascular support, but targeted inhibition of these pathways remains experimental in endometriosis [13,61,66]. In a benign chronic disease, even a strong biologic rationale is not sufficient unless the therapeutic index is acceptable for long-term use and consistent with fertility goals [61,66].

However, a section on endometriosis management should not stop at endocrine suppression alone. Pain in endometriosis is frequently multifactorial and may include nociceptive, neuropathic, and nociplastic components, which helps to explain why lesion burden does not always correlate with pain intensity and why some patients continue to report symptoms despite apparently adequate hormonal or surgical treatment [74,75]. Central sensitization has emerged as a clinically relevant contributor, particularly in patients with chronic pelvic pain, dyspareunia, dyschezia, and reduced quality of life. This means that persistent pain should not automatically be interpreted as treatment failure at the lesion level but may instead reflect altered pain processing [74].

For that reason, pain management should be framed as a multidisciplinary strategy, not merely as the adjunctive use of analgesics [75,76]. NSAIDs and other analgesics may be offered for symptom relief, either alone or together with hormonal therapy. However, evidence for their direct effect on disease progression is limited [55]. Pelvic floor dysfunction, myofascial pain, fear-avoidance behavior, and psychosocial distress may all contribute to the pain phenotype and should be addressed where relevant [77]. The available evidence for physiotherapy in endometriosis-specific populations remains limited. However, pelvic health physiotherapy may still be clinically useful in selected patients, especially when pelvic floor dysfunction, dyspareunia, or activity-related pain are present [55]. This broader framework is important because inflammatory mediators and neuroimmune interactions contribute to pain generation in endometriosis, yet direct cytokine-targeted therapy has not entered routine clinical practice [13,78].

Surgical management deserves more detailed discussion because it is one of the most important therapeutic pillars in patients with persistent pain, anatomical distortion, infertility, or organ compromise. Minimally invasive laparoscopy remains the preferred operative approach in most cases, both because it allows the direct visualization of disease and because it is associated with better postoperative recovery than open surgery [55,79]. Surgery is particularly relevant in ovarian endometriosis, deep infiltrating endometriosis, and cases with bowel, bladder, ureteral, or rectovaginal involvement, where disease burden may not be adequately controlled by medical treatment alone. Deep endometriosis involving the bowel or urinary tract often requires referral to experienced multidisciplinary centers, where gynecologic surgeons collaborate with colorectal, urologic, or thoracic specialists depending on lesion location [55,80]. The choice between excision and ablation should also be presented more precisely than as a generic surgical option. According to the ESHRE guideline, excision is generally preferred, especially in deep infiltrating disease, because it enables histologic confirmation and more complete removal of fibrotic or infiltrative lesions [55]. Ablation may still be acceptable in selected superficial lesions, but it is less suitable when the disease extends beneath the peritoneal surface. In practice, the decision should depend on lesion type, depth, localization, expected morbidity, and the surgeon’s expertise rather than on a one-size-fits-all hierarchy [55,79,81,82]. At the same time, surgery should not be described as curative. Even technically successful excision does not eliminate the risk of recurrence, and postoperative management often still requires hormonal suppression, pain-directed care, or fertility planning [55,56]. Surgical decision-making should therefore balance symptom severity, reproductive goals, prior treatment history, lesion location, and the likelihood that pain has central rather than purely peripheral drivers. This more nuanced framework is especially important in patients with repeated procedures, because repeated surgery may not meaningfully improve pain when sensitization mechanisms dominate the clinical picture [55,74].

Overall, contemporary management of endometriosis is best understood as a stepwise and phenotype-oriented strategy integrating hormonal suppression, individualized pain management, fertility planning, and surgery when clearly indicated [55,56]. Importantly, the therapeutic architecture in endometriosis is shaped not only by pathophysiology but also by the practical constraints of treating a benign chronic disease in many patients of reproductive age. This is precisely why some shared molecular targets have translated into standard therapy, whereas others remain investigational despite biological plausibility [61,64,66]. This perspective provides a more clinically relevant bridge to the comparison with endometrial cancer, where similar pathways are used within a very different therapeutic logic [58,59,61].

### 5.2. Management of Endometrial Cancer

Standard treatment includes surgery, radiotherapy, and systemic therapy. In developed countries, endometrial cancer represents the most prevalent malignant tumor of the female reproductive system [4]. In India, it is the second most common after cervical carcinoma [10,83]. Although most diagnoses occur in postmenopausal women, an estimated 15–25% of cases arise in those who have not yet reached menopause. Due to the absence of effective screening tools, timely and appropriate treatment at the point of diagnosis is essential [84]. The management of endometrial cancer typically involves a multimodal approach that includes surgical treatment, radiotherapy, chemotherapy, and, when indicated, hormone therapy [60]. Increasingly, this framework is further refined by molecular classification, which now has direct implications for prognosis, adjuvant therapy and selection of systemic treatment [58,59,85,86].

#### 5.2.1. Surgery

Primary treatment consists of total hysterectomy with bilateral salpingo-oophorectomy and staging procedures. Laparoscopy is favored for its lower complication rates, while vaginal hysterectomy limits staging options. Most cases are stage I, and adjuvant therapy is guided by surgical and pathological findings. The benefit of pelvic and para-aortic lymphadenectomy remains uncertain due to conflicting evidence [57,60]. Given that individuals with endometrial carcinoma are frequently of advanced age, obese, and affected by significant cardiovascular and metabolic comorbidities, surgical management should be carefully individualized to reduce perioperative risk. Performing nonessential interventions, such as lymphadenectomy, in patients without nodal involvement conveys no clinical advantage. It may predispose to both immediate and long-term morbidity [87]. The implementation of the sentinel lymph node biopsy (SLNB) approach in clinically stage I endometrial cancer has significantly limited the need for systematic pelvic and para-aortic lymphadenectomy. In the context of the present review, its main significance is that surgical and pathologic assessment defines disease extent and provides the basis for histologic and molecular risk stratification, which subsequently guides adjuvant treatment and clarifies when conservative management is not appropriate. 

#### 5.2.2. Adjuvant Radiotherapy

Patients with multiple high-risk features are the subgroup most likely to derive benefit from adjuvant therapy; however, they have been consistently underrepresented in clinical trials evaluating postoperative radiotherapy and chemotherapy. Randomized studies have established that the risk–benefit ratio does not support adjuvant treatment in low-risk endometrial cancer. Yet, these trials have generally been underpowered to determine its efficacy in patients with high-risk histopathologic or clinical features [88]. For patients who are considered medically inoperable due to comorbidities or other contraindications, radiation therapy represents a viable alternative for definitive management of endometrial cancer.

The current molecular classification of endometrial carcinoma, incorporated into FIGO staging in 2023, distinguishes four prognostic groups with direct implications for adjuvant treatment selection. Tumors with POLE exonuclease-domain mutations (POLEmut) are ultramutated, often high-grade endometrioid histology, yet they show excellent prognosis and may qualify for de-escalation or omission of adjuvant therapy in early stages [9,58,85]. MMR-deficient (MMRd) cancers, characterized by loss of mismatch repair proteins and microsatellite instability, have an intermediate prognosis and are candidates for radiotherapy and, in advanced or recurrent disease, immunotherapy; they are also important for identifying Lynch syndrome [9,58]. TP53-abnormal (p53abn) tumors, usually corresponding to copy-number high/serous-like subtype, confer the poorest prognosis and typically require intensified adjuvant chemoradiotherapy and consideration of targeted agents such as HER2 or PARP inhibitors in selected cases [58,59,85]. The residual group with no specific molecular profile (NSMP), largely copy-number low, microsatellite-stable, POLE-wild-type, and p53-wild-type, is the most frequent but biologically heterogeneous subgroup with intermediate, stage-dependent outcomes, for which additional markers (e.g., ER status, CTNNB1, 1q gain) are under investigation to refine risk stratification [58,89,90]. Together, this histomolecular framework enables restriction of adjuvant therapy to well-defined high-risk profiles while safely de-escalating treatment in favorable POLEmut disease [59,86]. For patients with early-stage endometrial cancer, current indications for adjuvant radiotherapy increasingly depend on the molecular classification of the tumor. International guidelines now encourage routine molecular profiling at diagnosis so that the molecular risk group is usually established on preoperative biopsy material and available to guide the choice and intensity of adjuvant radiotherapy before definitive surgical treatment is planned [91]. This is particularly evident in the current molecular classification of endometrial carcinoma, which refines prognosis and supports more individualized adjuvant management. The integration of molecular profiling into routine practice is clinically important here because it illustrates how endometrial cancer care is moving toward mechanism-based stratification, a concept also relevant to understanding the heterogeneity of endometriosis and its possible association with malignancy. 

#### 5.2.3. Chemotherapy

Adjuvant chemotherapy (CT) in endometrial cancer is mainly reserved for high-risk or advanced-stage disease. Surgery alone is usually sufficient for low-risk, early-stage patients. CT is generally not indicated for stage I-II endometrioid EC, except in high-risk cases. Stage III EC patients benefit from adjuvant CT. Radiation therapy may be added sequentially when indicated [92]. Cytotoxic chemotherapy remains the first-line treatment for most patients with metastatic or recurrent endometrial carcinoma. Single-agent anthracyclines, platinum compounds, and taxanes have demonstrated objective response rates above 20% in chemotherapy-naive individuals. Combination regimens generally achieve higher response rates, although they have not consistently improved overall survival. Phase III data support doxorubicin plus cisplatin as the Gynecologic Oncology Group (GOG) reference standard [93]. Adjuvant radiotherapy alone versus combined chemoradiotherapy has shown variable outcomes. Most recurrent or advanced cases receive first-line carboplatin plus paclitaxel. Second-line chemotherapy has limited efficacy, and the role of targeted therapies, including mTOR and antiangiogenic agents, remains investigational [94].

#### 5.2.4. Hormone Therapy of Endometrial Cancer

Hormonal therapy is a central, relatively low-toxicity option in hormone-sensitive endometrial cancer. It can preserve fertility in carefully selected early cases, palliate and sometimes control advanced/recurrent disease. Benefits and safety of the therapy depend on stage, grade, and ER/PR status, so individualized, specialist-led decisions are essential. Hormone therapy in endometrial cancer is used in three main settings: fertility-sparing treatment in young women, treatment of advanced/recurrent disease, and hormone replacement after surgery.

Oral progestins (MPA, MA) and/or levonorgestrel IUD achieve high complete response rates but have substantial recurrence risk; overall survival in carefully selected young women for fertility preservation remains excellent and similar to that for primary surgery [69,70]. Nevertheless, progestins, selective estrogen receptor modulators (SERMs), aromatase inhibitors, and GnRH analogs give response rates of ~10–35%, with the best results in low-grade, ER/PR-positive tumors [71,95]. In advanced or recurrent settings, hormonal therapy remains an option for selected low-grade, ER/PR-positive tumors because of its favorable toxicity profile, although responses are generally modest. This selective use underscores the importance of hormone responsiveness as a shared clinical and biological theme linking both diseases. 

#### 5.2.5. Hormone Replacement Therapy

Hormonal therapy in endometrial cancer is mainly administered orally (progestins, SERMs, aromatase inhibitors), as long-acting injectables (intramuscular or subcutaneous GnRH agonists and depot progestins), and locally via an intrauterine levonorgestrel-releasing system (LNG-IUS); topical ocular routes are not used in this indication [96].

Evidence suggests that women who have survived early-stage, low-grade endometrial cancer may safely use estrogen therapy to manage menopausal symptoms and mitigate the long-term effects of hypoestrogenism. Estrogen-only regimens are recommended, as the addition of progestins does not reduce the risk of cancer recurrence and may adversely affect breast tissue [97]. Adjuvant progesterone therapy is not indicated for routine prevention of endometrial cancer recurrence. Still, it may be considered in stage I patients seeking fertility preservation. Given that up to 30% of tumors initially diagnosed as grade I may be upgraded to final histopathology, patients should be counseled to undergo definitive hysterectomy upon completion of childbearing [60]. Available evidence suggests that carefully individualized estrogen-based therapy may be acceptable in appropriately selected survivors, whereas routine use for recurrence prevention is not supported. Counseling should remain individualized and take into account definitive pathology, recurrence risk, age, and reproductive plans.

#### 5.2.6. Role of Immunotherapy in Advanced Endometrial Cancer 

The landscape of immunotherapy in endometrial cancer is expanding, with several innovative combination strategies now under evaluation. Effective treatment planning requires stratification by immunologic subtype, specifically microsatellite instability-high (MSI-H) versus microsatellite stable (MSS) tumors, to guide future therapeutic approaches [98]. Immunotherapy combined with chemotherapy improves progression-free survival in advanced, metastatic, or recurrent endometrial cancer, including MMRd/MSI-H and MMRp/MSS subgroups. However, overall survival data are still immature. It may be considered as salvage therapy or in re-challenge strategies, particularly for patients with poor response markers. Immunotherapy also enables exploration of chemotherapy-sparing regimens to enhance quality of life. High costs and financial toxicity remain important considerations, underscoring the need for targeted, molecularly guided approaches to optimize outcomes [99,100]. Immunotherapy has become increasingly relevant in advanced, recurrent, and metastatic endometrial cancer, particularly when guided by mismatch repair or microsatellite instability status. For the present discussion, its importance lies less in therapeutic detail than in demonstrating how molecular characterization now directly shapes treatment selection and prognosis in endometrial cancer.

Therapeutic strategies for endometrial cancer are illustrated in Table 2.

### 5.3. Common Therapeutic Targets in Endometriosis and Endometrial Cancer

The overlap in hormonal, inflammatory and proliferative signaling between endometriosis and endometrial cancer raises an important translational question: if some of the same pathways are active in both diseases, why are they used so differently in clinical practice? The answer lies in the different goals of therapy. In cancer, treatment may accept substantial toxicity if it improves tumor control, delays progression, or prolongs survival. In endometriosis, treatment usually needs to be safe enough for prolonged use, compatible with fertility preservation and proportionate to a non-malignant disease burden [54,61,104]. A clear example is estrogen dependence. In both disorders, estrogen signaling contributes to cellular proliferation and disease maintenance [13,16]. However, endocrine therapy occupies a broader and more established role in endometriosis because long-term suppression of ovarian or local estrogen production can directly reduce lesion activity and pain [55,56,67,68]. In endometrial cancer, hormone therapy is used more selectively, mainly in fertility-sparing treatment of carefully chosen early-stage disease and in recurrent or advanced low-grade, hormone receptor-positive tumors [69,70,71,95]. Thus, the same biologic dependency on estrogen leads to different treatment algorithms because the expected endpoint differs: chronic disease suppression in endometriosis versus disease control in a defined oncologic subset in cancer [69,70,71,95,105]. A similar distinction applies to aromatase inhibition. In endometriosis, aromatase expression within ectopic lesions provides a rationale for the use of letrozole or anastrozole, particularly in refractory pain or selected infertility-related settings, however, these agents are not first-line because of hypoestrogenic adverse effects, effects on bone health and limited suitability for long-term use in young women [72,73]. In endometrial cancer, anti-estrogen strategies, including aromatase inhibitors, SERMs and other endocrine approaches, may produce clinically meaningful responses in selected ER/PR-positive, low-grade tumors, but overall activity is modest and remains restricted to well-selected clinical contexts [71,95,105].

The PI3K/AKT/mTOR axis provides another important example of shared biology with divergent therapeutic relevance. This pathway is frequently altered in endometrial cancer and contributes to proliferation, survival and treatment resistance, which explains the continued interest in mTOR-based and related targeted strategies in advanced or recurrent disease [85,92,106]. In endometriosis, activation of PI3K/AKT/mTOR signaling has also been implicated in lesion survival, angiogenesis, fibrosis and progesterone resistance, making this pathway biologically attractive as a future therapeutic target [13,61]. Nevertheless, mTOR inhibitors remain investigational in endometriosis because current evidence is limited, long-term safety is a major concern in a benign disease and adverse effects such as metabolic toxicity, mucositis and reproductive implications make routine use difficult to justify [61]. In contrast, these toxicities may be considered acceptable in advanced malignancy when progression control is the priority [107].

Angiogenesis illustrates the same principle even more clearly. Both endometriotic lesions and endometrial tumors depend on vascular support and VEGF-related signaling has been implicated in disease maintenance and progression in both settings [13,16,104]. Yet, antiangiogenic therapy has gained a more meaningful place in oncology than in endometriosis. In recurrent or persistent endometrial cancer, bevacizumab and other antiangiogenic strategies have shown activity, although their exact place remains limited and context-dependent rather than universal [66,92,104]. In endometriosis, by contrast, antiangiogenic treatment remains largely experimental. The reason is not lack of biologic rationale, but rather the problem of therapeutic index: inhibition of angiogenesis in reproductive-age women raises concerns related to ovarian physiology, implantation, wound healing and long-term tolerability, making aggressive antiangiogenic therapy difficult to justify for chronic benign disease [61,104].

Inflammatory cytokines represent a further area in which shared biology has not yet translated into symmetrical treatment. Cytokines such as TNF-α, IL-1 and IL-6 contribute to lesion persistence, pain generation and immune dysregulation in endometriosis, and inflammatory signaling also contributes to tumor progression and the microenvironment of endometrial cancer [13,108]. However, direct cytokine-targeted therapy has not become standard in either disease. In endometriosis, anti-TNF approaches generated early interest but have not shown sufficient clinical benefit to support routine use [108]. In endometrial cancer, inflammatory pathways are clinically relevant, but treatment has evolved more through biomarker-driven immunotherapy and combination systemic strategies than through isolated cytokine blockade [98,99,100]. This contrast highlights an important general principle: common molecular involvement does not automatically translate into a clinically useful drug target.

Taken together, these comparisons show why similar biology requires different therapeutic strategies. For endometrial cancer, treatment escalation is justified by the need for durable oncologic control. For endometriosis, however, long treatment duration, fertility goals, patient age and the need for acceptable tolerability set a much narrower boundary for targeted therapy [54,55,56,61]. At the same time, oncology still offers a useful conceptual framework for future endometriosis research. Molecular stratification, biomarker-guided treatment selection and a stronger distinction between disease phenotypes may help identify subsets of patient who could benefit from more mechanism-based therapies in the future. However, any such translation must remain cautious, because the threshold for toxicity in a benign chronic disorder is fundamentally different from that in cancer [61,104,107]. The comparison of therapeutic targets in endometriosis and endometrial cancer is presented in Table 3.

## 6. Prognosis

### 6.1. Prognostic Factors for Endometriosis

The prognosis of endometriosis is influenced primarily by disease phenotype, the extent of anatomical involvement, the risk of recurrence, and long-term symptom burden rather than by validated circulating biomarkers. In clinical practice, outcomes tend to be less favorable in patients with deep infiltrating endometriosis, multifocal disease, ovarian endometriomas, or lesions affecting the bowel, bladder, or uterus, as these phenotypes are associated with greater anatomical distortion, higher surgical complexity, and a greater likelihood of persistent or recurrent symptoms [55]. Disease stage alone does not always correlate directly with pain severity. Still, the depth of infiltration and the presence of deep endometriosis are clinically important because they are often linked to more complex symptoms, organ dysfunction, and the need for multidisciplinary care [79]. Another important prognostic issue is recurrence. Endometriosis is a chronic relapsing condition, and the risk of recurrence is influenced by lesion type, completeness of surgical excision, postoperative hormonal suppression, and duration of follow-up. Ovarian endometriomas and deep infiltrating lesions are particularly relevant in this context because they are associated with higher rates of repeated intervention and persistent symptoms in some patients [55,110]. The presence of atypical endometriosis may also be clinically relevant, not because it predicts the course of typical endometriosis in every patient, but because it is regarded as a histopathological variant with premalignant potential, particularly in relation to endometriosis-associated ovarian cancer. Although its prognostic significance in routine endometriosis care remains incompletely defined, the identification of atypia warrants careful pathological interpretation and follow-up [49]. Overall, prognosis in endometriosis should be considered in terms of symptom persistence, recurrence risk, fertility implications, and the burden of repeated treatment, rather than solely in terms of lesion presence. This perspective is more clinically meaningful because endometriosis behaves as a chronic, heterogeneous disease with variable long-term trajectories across patients [55].

### 6.2. Prognosis for Endometrial Cancer

Endometrial cancer prognosis is closely linked to FIGO stage, histological subtype, and molecular classification [72]. Stage I is subdivided based on tumor aggressiveness, the depth of myometrial invasion, lymphovascular space invasion (LVSI), and concomitant low-grade ovarian involvement, with nonaggressive tumors confined to the endometrium or limited myometrium (IA1–IA3) generally carrying a favorable prognosis [105]. More extensive myometrial involvement (IB) or aggressive histological types without myometrial invasion (IC) indicate a higher risk. Stage II prognosis varies according to cervical stromal infiltration, substantial LVSI, or aggressive histology. Stage III outcomes are influenced by adnexal or serosal involvement, vaginal/parametrial extension, pelvic peritoneal metastases, and nodal status, including micrometastases and macrometastases. Stage IV disease, with bladder/rectal infiltration, extrapelvic peritoneal spread, or distant metastasis, carries the poorest prognosis. Incorporating molecular classification (POLEmut, MMRd, NSMP, p53abn) further refines prognostic assessment, with certain molecular subtypes prompting upstaging or downstaging even in early-stage disease [72,73,106].

## 7. Future Research

### 7.1. Noninvasive Biomarkers and Precision Medicine Approaches

The accurate diagnosis and effective management of endometriosis and endometrial cancer remain challenging due to the invasiveness of current diagnostic procedures and the heterogeneity of disease presentation [111]. The development of noninvasive biomarkers could facilitate earlier detection, enable monitoring of disease progression, and support risk stratification, while precision medicine approaches have the potential to tailor therapies to individual molecular and clinical profiles, ultimately improving outcomes and quality of life for patients [112]. A transcriptomics-guided approach has highlighted several promising biomarkers for fluorescent imaging, paving the way for enhanced intraoperative detection and treatment of endometriosis [113,114]. Emerging evidence also suggests that serum biomarkers and circulating miRNAs could offer less invasive and potentially more cost-effective tools for diagnosis, postoperative monitoring, and treatment stratification [110]. However, current data on biomarkers, including HSD17B2, BCL-6, NO, PR isoforms, RASSF family members, GATA factors, ER isoforms, VEGF, IL-6, MMP-9, CA-125, and multiple miRNAs, remain limited by small cohorts and methodological heterogeneity, currently precluding their routine clinical application as reliable prognostic or predictive tools [72,105]. Standardization of study design and validation in large prospective cohorts will be necessary before such markers can be integrated into precision medicine approaches for endometriosis [72,105].

### 7.2. Potential Molecular Targets and Novel Therapies 

Emerging therapeutic strategies for endometriosis and endometrial cancer are increasingly focused on personalized approaches targeting specific pathogenic pathways. In endometrial cancer, traditional surgery and adjuvant therapy remain standard, but advanced or metastatic cases often show limited responsiveness, prompting investigation into molecularly targeted treatments [115]. Agents modulating signaling pathways such as EGFR, VEGFR, and PI3K/PTEN/AKT/mTOR have demonstrated modest efficacy, particularly when combined with chemotherapy or radiotherapy. Immunotherapy and exploration of the tumor immune microenvironment are shaping new strategies, with ongoing trials evaluating checkpoint inhibitors to overcome resistance mechanisms [16]. For endometriosis, current medical treatments, including NSAIDs, progestins, oral contraceptives, GnRH agonists/antagonists, and aromatase inhibitors, primarily provide symptomatic relief, yet their long-term efficacy is limited. Novel hormonal modulators, such as selective progesterone receptor modulators (e.g., Proellex) and next-generation GnRH antagonists (e.g., elagolix), show promise in controlling disease progression and alleviating pain. However, side effects such as bone density loss or hepatic toxicity must be monitored. Additional molecular approaches under investigation target estrogen and progesterone pathways, receptor responsiveness, epigenetic modifications, and angiogenesis, including VEGF inhibitors like bevacizumab [61]. While preclinical studies and early-phase trials indicate potential, high-quality clinical evidence remains limited, highlighting the need for further research into these innovative therapies to improve outcomes and quality of life [16,116].

## 8. Conclusions

Endometriosis and endometrial cancer are distinct gynecological conditions that share key biological mechanisms, including chronic inflammation, hormonal dysregulation, and overlapping molecular pathways. Epidemiological data indicate a modest association between endometriosis and an increased risk of endometrial cancer. However, this relationship may be influenced by detection bias and confounding metabolic factors. Advances in molecular classification and precision medicine have improved risk stratification and therapeutic decision-making, particularly in endometrial cancer management. In endometriosis, emerging molecular insights may support earlier diagnosis and more targeted, noninvasive treatment strategies. Addressing modifiable metabolic and inflammatory risk factors represents a potential avenue for prevention across both conditions. From a therapeutic perspective, the integration of individualized surgical planning, optimized hormonal therapy, and, where appropriate, targeted and immunomodulatory treatments may improve long-term outcomes while minimizing treatment-related morbidity. Further large-scale, prospective studies and the development of reliable biomarkers are essential to clarify this association and translate mechanistic insights into optimized, individualized patient care.

## Figures and Tables

**Table 1:** Shared and Distinct Biological Features of Endometriosis and Endometrial Cancer [5,51,52,53,54].

Feature	Endometriosis	Endometrial Cancer
Pathological character	Chronic estrogen-dependent inflammatory disease characterized by ectopic endometrial-like tissue	Estrogen-driven malignant neoplasm arising from the endometrial lining
Hormonal milieu	Estrogen excess and progesterone resistance promote lesion survival and proliferation	Prolonged unopposed estrogen exposure promotes endometrial hyperplasia and malignant transformation
Inflammatory profile	Persistent local and systemic inflammation with elevated cytokines, chemokines, and immune dysregulation	Tumor-associated inflammatory microenvironment supports proliferation, invasion, and immune escape
Cellular behavior	Increased proliferation, invasion, adhesion, angiogenesis, and resistance to apoptosis in ectopic lesions	Uncontrolled proliferation, invasive growth, angiogenesis, and apoptosis evasion in malignant cells
Molecular alterations	Dysregulated signaling pathways and epigenetic changes; cancer-associated mutations may occur in some lesions	Frequent genomic and molecular alterations involving PI3K/AKT, PTEN, PIK3CA, KRAS, ARID1A, and mismatch repair pathways
Metabolic associations	Linked to oxidative stress, altered immune metabolism, and chronic inflammatory burden	Strongly associated with obesity, insulin resistance, metabolic syndrome, and adipose tissue inflammation
Microenvironment	Estrogenic, inflammatory, and pro-angiogenic niche facilitating persistence of ectopic implants	Pro-tumorigenic microenvironment promoting malignant progression and metastatic potential
Potential mechanistic overlap	Shares inflammatory, hormonal, epigenetic, and proliferative mechanisms with endometrial carcinogenesis	May arise in a biological context shaped by chronic inflammation, excess estrogen exposure, and metabolic dysfunction
Clinical presentation	Pelvic pain, dysmenorrhea, dyspareunia, and infertility	Abnormal uterine bleeding, especially postmenopausal bleeding
Implication for disease link	Considered a potential risk-modifying condition, although the association is modest and may be influenced by detection bias	Represents the malignant endpoint potentially favored by overlapping hormonal, inflammatory, and metabolic pathways

**Table 2:** Overview of Therapeutic Strategies Used for Treating Endometrial Cancer [101,102,103].

Therapeutic Modality	Main Interventions	Indications	Key Limitations and Considerations
Surgery	Total hysterectomy with bilateral salpingo-oophorectomy, surgical staging; SLNB, selective lymphadenectomy	Primary treatment, especially for early-stage disease	Laparoscopy preferred; benefit of lymphadenectomy uncertain; treatment should be individualized due to age, obesity, and comorbidities
Radiotherapy	Adjuvant or definitive radiotherapy	Patients with high-risk features or those medically inoperable	Limited evidence in high-risk subgroups; not recommended for low-risk disease
Chemotherapy	Adjuvant chemotherapy; first-line systemic therapy (carboplatin + paclitaxel); combination regimens	High-risk, stage III, advanced, metastatic, or recurrent disease	Limited benefit in early-stage low-risk disease; second-line therapy has low efficacy
Hormone replacement therapy	Estrogen-only therapy; progesterone therapy for fertility preservation	Early-stage, low-grade survivors; selected stage I patients	Not indicated for routine recurrence prevention; definitive surgery recommended after childbearing
Immunotherapy	Immune checkpoint inhibitors alone or combined with chemotherapy	Advanced, metastatic, or recurrent disease (MSI-H/MMRd and MSS/MMRp)	Overall survival data immature; high cost and financial toxicity

**Table 3:** Comparison of common therapeutic targets in endometriosis and endometrial cancer [55,56,61,67,68,69,70,71,95,104,107,108,109].

Target	Role in Endometriosis	Role in Endometrial Cancer	Drugs/Classes Used	Key Limitations and Considerations
Estrogen receptor (ER) signaling	Drives lesion persistence, inflammation, and pain, central therapeutic axis in long-term suppression	Promotes growth in hormone-responsive tumors, relevant mainly in selected low-grade, ER/PR-positive disease	Combined hormonal contraceptives, progestins, GnRH agonists/antagonists in endometriosis, progestins, SERMs, endocrine therapy in selected cancer cases	In endometriosis, prolonged suppression must remain compatible with quality of life and fertility, in cancer, hormonal therapy is limited to selected molecular and clinicopathologic subgroups [55,56,57,58,83,84,85,86,97]
PI3K/AKT/mTOR pathway	Implicated in lesion survival, fibrosis, angiogenesis, and progesterone resistance, promising but still investigational target	Frequently dysregulated, associated with proliferation, survival, and therapeutic resistance, especially in advanced/recurrent disease	Experimental mTOR inhibitors in endometriosis, mTOR/PI3K/AKT-directed strategies in recurrent/advanced cancer	In endometriosis, toxicity and need for long-term treatment limit clinical applicability, in cancer, higher toxicity may be acceptable when progression control is the goal [95,99]
Aromatase	Supports local estrogen biosynthesis within lesions, relevant in refractory disease	Supports endocrine responsiveness in selected hormone-sensitive tumors	Letrozole, anastrozole	In endometriosis, not first-line because of hypoestrogenic adverse effects and bone loss risk, in cancer, responses are modest and mainly seen in selected ER/PR-positive tumors [96,97,98]
Angiogenesis/VEGF	Important for implantation and maintenance of ectopic lesions	Supports tumor growth, invasion, and metastatic spread	Experimental antiangiogenic agents in endometriosis; bevacizumab and other antiangiogenic strategies in selected recurrent/advanced cancer settings	In endometriosis, fertility, ovarian physiology, implantation, and long-term safety are major barriers, in cancer, toxicity may be justified but benefit remains context-dependent [95,100]
Inflammatory cytokines (e.g., TNF-α, IL-6, IL-1)	Contribute to pain, lesion survival, immune dysregulation, and chronic inflammation	Shape tumor microenvironment and may contribute to progression and treatment response	NSAIDs for symptom relief in endometriosis, experimental cytokine-directed approaches, immunotherapy-based systemic strategies in cancer	Cytokine blockade has not become standard in endometriosis due to limited efficacy, in cancer, inflammation is clinically important but usually targeted indirectly through broader systemic or immune-based approaches [55,89,90,91,101]

## Data Availability

No new data were created or analyzed in this study. Data sharing is not applicable to this article.
